# Health-Promoting Properties of Processed Red Cabbage (*Brassica oleracea* var. *capitata* f. *rubra*): Effects of Drying Methods on Bio-Compound Retention

**DOI:** 10.3390/foods13060830

**Published:** 2024-03-08

**Authors:** Nicol Mejías, Antonio Vega-Galvez, Luis S. Gomez-Perez, Alexis Pasten, Elsa Uribe, Anielka Cortés, Gabriela Valenzuela-Barra, Javiera Camus, Carla Delporte, Giuliano Bernal

**Affiliations:** 1 Departamento de Ingeniería en Alimentos, Universidad de La Serena, Raúl Bitrán 1305, La Serena 1700000, Chile; 2 Instituto Interdisciplinario de Investigación y Postgrado, Universidad de La Serena, Raúl Bitrán 1305, La Serena 1700000, Chile; 3 Laboratorio de Productos Naturales, Facultad de Ciencias Químicas y Farmacéuticas, Universidad de Chile, Santiago 8380000, Chile; 4 Laboratorio de Biología Molecular y Celular del Cáncer, Departamento de Ciencias Biomédicas, Facultad de Medicina, Universidad Católica del Norte, Larrondo 1281, Coquimbo 1781421, Chile; gbernal@ucn.cl

**Keywords:** anti-inflammatory, antioxidants, antiproliferative, bio-compounds, drying, red cabbage

## Abstract

The aim of this work is to describe the effect of convective drying (CD), vacuum drying (VD), infrared drying (IRD), low-temperature vacuum drying (LTVD) and freeze drying (FD) on bio-compound retention of red cabbage and its beneficial health properties. The total phenolics content (TPC), flavonoids (TFC), anthocyanin (TAC) and glucosinolates (TGC) were determined by spectrophotometry. The profiles of phenolic acids, amino acids and fatty acids were determined by HPLC-UV-DAD, LC-DAD and GC-FID, respectively. Antioxidant potential was verified by DPPH and ORAC assays. The antiproliferative activity was measured in the human gastric cell line (AGS). Anti-inflammatory activity was evaluated by phorbol 12-myristate 13-acetate and arachidonic acid models. VD showed high values of TPC = 11.89 ± 0.28 mg GAE/g d.m.; TFC = 11.30 ± 0.9 mg QE/g d.m.; TAC = 0.265 ± 0.01 mg Cya3glu/g d.m.; and TGC = 51.15 ± 3.31 µmol SE/g d.m. Caffeic acid, ferulic acid and sinapic acid were identified. The predominant amino acid and fatty acid were glutamic acid and γ–linolenic acid, respectively. The antioxidant potential was dependent on drying methods for both DPPH and ORAC assays. Dried red cabbage extracts showed clear anti-inflammatory and antiproliferative activity. The dehydration process is an alternative for the retention of bio-compounds and health-promoting properties of red cabbage.

## 1. Introduction

Globally, people are becoming increasingly concerned about health and nutrition [1], intensifying the demand for and consumption of healthy foods. Fruits, vegetables and their derivatives have been reported as important natural sources of bio-compounds, such as vitamins, minerals, polyunsaturated fatty acids (ω-3, ω-6), polyphenolic compounds, amino acids, sulfur compounds, polysaccharides and enzymes [2]. These compounds might have an impact on the physiological or cellular activities of the humans who consume them, because they promote beneficial health properties, such as antioxidant [3], anti-inflammatory [4], antidiabetic, anti-obesity [5], anti-cancer [6] and neuroprotective properties [7], and protect against degenerative diseases such as cardiovascular diseases, Acquired Immuno Deficiency Syndrome (AIDS) and neurological diseases (Parkinson’s disease, muscular dystrophy, Alzheimer’s disease, multiple sclerosis and amyotrophic lateral sclerosis) among others [8].

Red cabbage (*Brassica oleracea* var. *capitata* f. *rubra*), also called purple cabbage, originated in Europe and nowadays is produced and harvested all over the world [9]. This cruciferous vegetable is a natural source of phytochemicals with important content of vitamins C, K and A [10,11], anthocyanin [12], fatty acids, fiber [13], phenolic compounds [14], flavonoids [15] and glucosinolates [16], among others. Glucosinolates (GLS) are a very important group of sulfur-containing secondary plant metabolites and they are especially present in red cabbage [17]. Their positive effects on human health are ascribed to isothiocyanates, being GLS hydrolysis products, since when plant tissues undergo damage due to chewing, harvesting, post-harvest processing or pest activity, the enzyme myrosinase acts on GLS to form these metabolites [18]. On the other hand, several studies have reported some beneficial health properties of red cabbage, which might be explained by its bio-compounds, such as polyphenols and GLS hydrolysis products. In this sense, Koss-Mikołajczyk et al. (2019) [16] described the α-amylase and α-glucosidase inhibition of red cabbage extracts related to an antidiabetic effect, and antioxidant potential measured by the FRAP [19] and DPPH assays [20] has also been reported. Other relevant functional properties such as anti-cancer [21], antimicrobial activity [22] and anti-inflammatory activity [23] have been found in red cabbage extracts as well. In addition, red cabbage extract has been shown to exhibit enhanced antibacterial and antiproliferative activity compared to other cruciferous vegetables like cauliflower, white cabbage and Chinese cabbage [24]. Furthermore, the consumption of red cabbage microgreens can prevent hypercholesterolemia by reducing triacylglycerol levels and inflammatory cytokine expression [25]. The consumption of red cabbage involves the use of thermal processes, mainly domestic boiling, which leads to the degradation of heat-sensitive bio-compounds and therefore entails a loss of health-promoting functional activity [18]. Thus, the challenge is to increase the shelf life of this cruciferous vegetable through a process that retains these bio-compounds and their properties. 

On the other hand, drying is an ancient and useful alternative to food conservation by water removal. Moisture removal from fresh products prevents the development of microorganisms, which allows an increase in shelf life [26]. Drying methods make it possible to preserve and stabilize fresh fruits and vegetables with high moisture content. 

Hot air drying stands out as one of the most commonly used methods for drying agricultural products due to its simplicity and cost-effectiveness. However, it may induce color changes, texture hardening and nutrient loss due to high temperatures. In contrast, vacuum drying operates in an oxygen-deprived environment, effectively preserving color and flavor and minimizing oxidation-related losses. Nevertheless, its reliance on specialized equipment increases operational costs. Microwave drying, characterized by lower energy consumption and shorter drying times, offers potential advantages. However, its applicability is limited to heat-sensitive products and may result in uneven drying and quality deterioration [27]. Low-temperature vacuum drying emerges as an excellent alternative for producing high-quality products, as it preserves color, flavor and nutritional properties while also offering energy savings compared to techniques like freeze drying [28]. Infrared drying can achieve higher drying rates compared to other drying methods, improving efficiency and shortening process time, making it a promising method for obtaining top-quality dried products [29], but it is important to consider its weak penetrative ability and the risk of overheating and burning, which can negatively affect final quality [30]. Freeze drying is characterized by its high-quality products, avoiding bio-compound oxidation with minimal loss after process. Regrettably, it is an expensive process [27]. 

Studies have been reported on similar raw materials, such as Chinese cabbage, that were dehydrated by different methods such as oven drying, solar cabinet drying, sun drying, and freeze drying, showing a greater impact on phenolic compounds [31]. Studies on red cabbage dehydrated by hot air drying, microwave drying, vacuum drying, vacuum microwave drying and vacuum freeze drying have concluded that freeze drying maintains the content of phenolic compounds better [32]. However, this methodology involves greater energy consumption, increasing costs, and has a greater environmental impact. There are few works on the dehydration of red cabbage; however, existing studies primarily focus on assessing its nutritional content, particularly anthocyanins and physical properties, and the most common drying techniques explored include hot air-, vacuum- and microwave drying [32,33,34], but there is still a need for further research on the impact of different drying methods on the retention of specific bioactive compounds and their potential health benefits.

Applying drying technologies to food products leads to various changes at the cellular level, affecting morphological (shrinkage, cell shape, plasmolysis or lysis), structural (loss of water, cell size, shape and thickness, cracks, fissures and porosity), physicochemical (denaturation of proteins, lipid degradation and oxidation, Maillard reaction products) and nutritional aspects (decreased moisture content, macromolecule concentrations, variable bioaccessibility of nutrients, changes in antioxidant activity) [35]. 

On the other hand, the conditions under which these processes are applied can help improve the structural changes, quality and retention of bio-compounds with health-promoting properties.

The aim of this work is to describe the effect of five drying methods: convective drying (CD), vacuum drying (VD), infrared drying (IRD), low-temperature vacuum drying (LTVD) and freeze drying (FD) on the retention of bio-compound contents of red cabbage and their beneficial health properties, such as antioxidant, antiproliferative and anti-inflammatory. 

## 2. Materials and Methods

### 2.1. Solvents and Reagents

All reagents used were of analytical grade and were purchased from SIGMA Aldrich (St. Louis, MO, USA): methanol (MeOH), (0.1%) formic acid, sodium hydroxide (NaOH), hydrochloric acid (HCl), Folin–Ciocalteu reagent, (20%), sodium carbonate solution (Na_2_CO_3_), (5%) sodium nitrite solution (NaNO_2_), (10%) aluminum trichloride solution (AlCl_3_), sodium tetrachloropalladate II (Na_2_PdCl_4_) reagent, Sinigrin (Sng), acetonitrile (C₂H₃N), borate buffer solution, boron trifluoride (BF_3_), Sodium chloride (NaCl), o-phthalaldehyde (OPA), dichloromethane (CH_2_Cl_2_), 6-Hydroxy-2,5,7,8-tetramethylchoman-2-carboxylic acid (Trolox), 2,2-diphenyl-1-picrylhydrazyl (DPPH), 2,2-azobis(2-amidinopropane) dihydrochloride (AAPH), gallic acid, quercetin, arachidonic acid (AA, C_20_H_32_O_2_), phorbol 12-myristate 13-acetate (TPA), acetone (CH₃CH₃), F-12K medium (2 mM L-glutamine and 1500 mg/L NaHCO_3_, Corning^®^, Corning, NY, USA), Dulbecco’s modified Eagle’s medium (DMEM) (Corning^®^, Corning, NY, USA), fetal bovine serum (FBS) (Hyclone, Logan, UT, USA), penicillin and streptomycin (Corning^®^, Corning, NY, USA).

### 2.2. Raw Material and Drying Methods

#### 2.2.1. Raw Material

Fresh red cabbage (*Brassica oleracea* var. *Capitata rubra*) was purchased from a local market in La Serena city, Chile. Visibly damaged red cabbage leaves were discarded and the remaining leaves were washed and cut into 1 × 1 cm^2^ pieces. Blanching was applied to the chopped cabbage according to Tao et al. (2019) [36]. The different batches were submerged in boiled water for 30 s and then quickly cooled in an ice–water mixture. The surface water was drained and subsequently dried with absorbent paper. Finally, the batches were subjected to dehydration by five different drying methods.

#### 2.2.2. Drying Procedure

The blanched red cabbage batches were subjected to five drying techniques: convective drying (CV), vacuum drying (VD), infrared drying (IRD), low-temperature vacuum drying (LTVD) and freeze drying (FD). The individual drying conditions were determined on the basis of the pre-experiments. Table 1 shows the process conditions for all methods. 

Once the dehydrated samples were obtained, they were then pulverized with a basic analytical mill (IKA A-11, Wilmington, DE, USA), sieved with a sieve with a maximum pore size of 1000 microns and immediately packed in closed plastic bags and stored at 4 °C for further analysis. 

### 2.3. Proximate Composition 

A proximate composition analysis of red cabbage was performed by AOAC (Association of Official Analytical Chemists, 1990) [37] methodologies. The moisture content was determined through a gravimetric determination of moisture by heating the sample until it reached a constant weight; fat (960.39) was determined by Soxhlet continuous extraction with petroleum ethers; ash (923.03) was determined based on a gravimetric determination after combustion of the sample at 550 °C; crude protein (992.15) was determined by Kjeldahl digestion with sulfuric acid, followed by distillation and titration; and crude fiber (978.10) was determined based on sequential digestion with acid and alkali solutions to remove proteins and soluble carbohydrates. The water activity (a_w_) for each sample was determined at 25 °C in a water activity meter (AquaLab 4 TE, Pullman, WA, USA).

### 2.4. Assessment of Bio-Compounds

#### 2.4.1. Extraction Methods 

The extraction of the antioxidant compounds was performed by a solvent extraction method based on the work of Aghajanzadeh (2014) [38] with some differences. In total, 10 g of fresh-blanched (fresh-b) sample and 4 g of dry sample (the quantities were selected based on preliminary studies) were taken, extracted under continuous shaking (Orbital Shaker, OS-20, BOECO, Hamburg, Germany) at 250 rpm for 1 h at room temperature, initially with 15 mL of an aqueous methanol solution (80%). Then, each tube was subjected to centrifugation (5000 rpm, 20 min, 4 °C). The supernatants were collected and filtered and subsequently concentrated to dryness in a multi-evaporator (Büchi P-6, Flawil, Switzerland) at 40 °C. After that, they were reconstituted to a volume of 5 mL with methanol/formic acid (99:1 *v*/*v*).

The extraction method for HPLC-UV-DAD was carried out according to Lee et al. (2018) [39]. Briefly, 300 mg of sample was mixed with 9 mL of 53% aqueous methanol (*v*/*v*, containing 2.6 M NaOH) and incubated at room temperature on an orbital shaker. After 20 h, the pH of the solution was adjusted to pH 2 with HCl and centrifuged at 5000 rpm at 10 °C for 10 min. The supernatant was filtered (#1 filter, Whatman International Limited, Kent, UK) and the final volume was put into a 10 mL volumetric flask with a 53% methanol aqueous solution prepared previously. All samples were filtered with 0.45 µm polypropylene filters and then injected into an HPLC system.

#### 2.4.2. Determination of Total Phenolics, Total Flavonoids and Total Anthocyanin Content

Total phenolics content (TPC) was determined according to Uribe et al. (2022) [40] with modifications. Overall, 15 µL of red cabbage extract was mixed with 100 µL of Folin–Ciocalteu reagent (0.2 N) in a microplate. Then, 100 µL of sodium carbonate solution (60 mg/mL) was added and incubated at room temperature in the dark for 90 min. After incubation, TPC absorbance was read at 750 nm using a multiplate reader (Perkin—Elmer, Victor TM X3, Hamburg, Germany). Gallic acid (0.02–0.6 mg/mL) was used as a standard to perform the calibration curve (y = 4.4392x − 0.065; R^2^ = 0.9978). The results were expressed as mg gallic acid equivalent (GAE)/g dry matter (d.m.). All measurements were performed in triplicate.

Total flavonoid content (TFC) was measured by a colorimetric assay according to Dini et al. (2010) [41]. For this purpose, 0.5 mL of red cabbage extract was combined with 2 mL of distilled H_2_O. The reaction was initiated by the addition of 0.15 mL of 5% NaNO_2_. After 5 min, 150 µL of 10% AlCl_3_ solution was added and allowed to stand for 6 min at room temperature. Then, 1.0 mL of a 1 M NaOH solution and 1.2 mL of deionized water were added. Quercetin was used to perform the standard curve (y = 0.0017x − 0.0043; R^2^ = 0.9976). Quercetin equivalent (QE)/g d.m. was the unit used to express the TFC. 

The pH differential approach was used to calculate the total anthocyanin content (TAC) [42]. The samples were diluted with pH 1.0 potassium chloride 0.25 M and pH 4.5 sodium acetate 0.4 M buffers. For every buffer, the absorbance was measured at 510 and 700 nm. TAC (expressed in terms of cyanidin-3-glucoside) was determined using the following equations:

(1) $$A=\left(A_{510}-A_{700}\right)pH_{1.0}-\left(A_{510}-A_{700}\right)pH_{4.5}\;\;\;\;$$

(2) $$TAC=\left(A\times MW\times DF\times V_{e}\times 1000\right)/\left(Ɛ\times 1\times M\right)$$

where MW is 449 g/mol (molecular weight of cyanidin-3-glucoside), DF the dilution factor, $V_{e}$ the volume of extract, Ɛ the molar extinction coefficient of cyanidin-3-glucoside (26,900) and M the mass of extracted red cabbage.

#### 2.4.3. Determination of Total Glucosinolates

The total glucosinolate content was determined according to Aghajanzadeh et al. (2014) [38]’s methodology, based on a reaction with sodium tetrachloropaladate II (Na_2_PdCl_4_) 2 mM (58.8 mg sodium tetrachloropaladate + 170 µL concentrated HCl + 100 mL double-distilled water). The reaction mixture containing 60 µL of extract and 1800 µL of Na_2_PdCl_4_ 2 mM was incubated at room temperature for 30 min in the dark and the absorbance of the developed color was read at 450 nm. Sinigrin (Sigma-Aldrich, St. Louis, MO, USA, S1647) was used as an internal standard for a calibration curve (y = 0.084*x* − 0.0286, R^2^ = 0.9983), between 0.02 and 0.98 mg/mL. Values were expressed as µmol sinigrin equivalent/g d.m. All measurements were performed in triplicate.

#### 2.4.4. Determination of Phenolic Acids Profile

Phenolic acid profiles were analyzed by a high-performance liquid chromatography–ultraviolet detection (HPLC-UV) system (Agilent 1200 series, Santa Clara, CA, USA) equipped with a diode array detector (DAD) and controlled with the ChemStation software B.04.02(96). We employed the chromatographic conditions stated by López et al. (2017) [43]. Phenolic acids were separated on a Kromasil 100-5C18 column (250 ± 4.6 mm; Eka Chemical, Bohus, Sweden) using a binary gradient consisting of solvents A (0.1% formic acid in methanol, pH 2.6) and B (100% acetonitrile) at a flow rate of 0.7 mL/min at 25 °C. The gradient of solvent B was 13% at 0 min, 55% at 18 min, 60% at 23 min and 13% at 25 min (initial conditions). Spectral data were recorded at 310 nm. The phenolic acids in all samples were analyzed by comparing their retention times and UV spectra with authentic standards. The quantified phenolic acids were expressed as µg/g d.m. The used solvents were of HPLC analytical grade and were obtained from Merck (Merck KGaA, Darmstadt, Germany), and the standards were obtained from Sigma (St. Louis, MO, USA).

#### 2.4.5. Determination of Amino Acid Content and Fatty Acids Profile

For fatty acids profile, extraction and methylation of samples was based on the methodology outlined by Folch et al. (1957) [44]. A 14% boron trifluoride–methanol solution (BF_3_-MeOH) was used to transform lipids into fatty acid methyl esters (FAMEs). Subsequently, FAME extraction was carried out by incorporating hexane and rinsing with 20% NaCl. The organic fraction was then recovered, evaporated to dryness, and reconstituted in 1 mL of hexane. Quantification of FAMEs was achieved using gas chromatography (GC) equipment (Clarus 600 FID model, PerkinElmer, Waltham, MA, USA) with a flame ionization detector (GC-FID) and an Omega Wax 320 capillary column (30 m × 0.320 mm × 0.25 μm, Supelco, St. Louis, MO, USA) with temperature limits set at 20–250 °C. The temperature ramp was first maintained at 60 °C for three minutes, and then it rose by 10 °C every minute to reach 260 °C at a flow rate of 1.0 mL/min (carrier gas: nitrogen). Quantification of individual fatty acids was achieved by comparing the retention times and peak areas of the FAME standard (Supelco 37 Component FAME Mix, Sigma, n° CRM47885, St. Louis, MO, USA) with the unknown sample.

On the other, an HPLC pre-column derivatization technique was used to perform the amino acid profile study [45]. First, 200 mg of sample was placed on semi-capped hydrolysis tubes, and then 10 mL of 6 N HCl solution was added, followed by a 24 h incubation in an oven at 120 °C. The resulting solution was then transferred to a volumetric flask filled with distilled water up to 50 mL. An aliquot of 100 μL was extracted, pH-adjusted to 10.0 with borate buffer, concentrated to dryness, reconstituted to a volume of 200 μL with borate buffer (pH 10.0) and filtered using a 0.22 μm Nylon syringe filter in preparation for derivatization. Amino acids were pre-column derivatized using the Jasco AS-2055 autosampler and OPA. Analysis was conducted on an Agilent HPLC system with a ZORBAX Eclipse AAA amino analysis column (3.5 μm, 4.6 × 150 mm) at 40 °C. The mobile phase consisted of borate buffer (pH 7.8; A), acetonitrile–methanol–water (90:90:10, *v*/*v*/*v*; B) and 100% methanol (C) at 2 mL/min. Gradient elution: 0–1.9 min, 100% A; 18.1–18.6 min, 42% A, 58% B; 22.3 min, 30% A, 70% B; 22.40–26.00 min, 100% C; 26.10–28.00 min, 100% A. Detection was performed at 338 nm within the range of 240 nm to 400 nm. 

#### 2.4.6. Determination of Sulforaphane (SFN) Content

A calibration curve for sulforaphane content was established using a standard range of 0.01 to 0.98 mg/mL, dissolved in dichloromethane, evaporated, and reconstituted with acetonitrile to maintain the original concentration. Five grams of dried cabbage were diluted with 40 mL of Milli-Q water and incubated at 37 °C with 200 rpm agitation for 3 h. The resulting sample was introduced into a separating funnel with 10 mL of dichloromethane and carefully shaken, and the organic fraction was collected. This process was repeated twice. The solid phase was collected and centrifuged for 5 min at 10,000 rpm. The organic phase was collected, evaporated and reconstituted with 1 mL of acetonitrile. The reconstituted fraction was filtered (0.45 µm) and injected into an HPLC system (Waters Alliance e2995, Detector DAD 2996, Empower 3) equipped with a Waters Spherisorb ODS 2, 5 µm, 4.0 × 250 mm column. The mobile phase consisted of (A) 0.05% phosphoric acid in acetonitrile/water (5:95) and (B) 0.05% phosphoric acid in acetonitrile/water (95:5) at a flow rate of 1 mL/min. The injection volume was 20 µL. All samples were prepared protected from light, and once prepared, they were immediately analyzed. Results were expressed as mg SFN/mL.

### 2.5. Health-Promoting Properties

#### 2.5.1. Antioxidant Potential by DPPH and ORAC Assays

The determination of antioxidant capacity by DPPH assay was performed according to the method described by Grajeda-Iglesias et al. (2016) [46] with some modifications. A total of 20 µL of red cabbage extract was taken and 180 µL of DPPH solution (120 µM DPPH in 80% methanol freshly prepared before assay) was added in 96-well transparent microplates and allowed to react for 30 min in a multiplate reader (Perkin—Elmer, Victor TM X3, Hamburg, Germany). Trolox was used as a standard to perform a calibration curve from 1 to 500 µM Trolox (y = −0.0014x + 0.6844; R^2^ = 0.9914). The absorbance was read at 510 nm and the values were expressed as µmol TE/g d.m.

The ORAC assay methodology was followed according to Uribe et al. (2014) [47]. In a 96-well microplate reader (Perkin Elmer, Victor X3, Hamburg, Germany), 50 μL of red cabbage extract and 40 μL of phosphate buffer (pH 7.4) were combined. After adding 200 μL of fluorescein (100 nM) to each well, the mixture was incubated for 20 min at 37 °C. Then, 35 μL of a 0.36 M solution of AAPH was added to each well for readings at excitation (λ_ex_) and emission (λ_em_) wavelengths at 485 nm and 535 nm, respectively. Plotting the Trolox concentrations ranging from 5 to 250 μM against the fluorescence decay curve (area under the curve) yielded the calibration curve for the ORAC assay, which was then represented by the following equation: *y* = −0.0012x + 0.5901 (R^2^ = 0.9808). Trolox equivalent (µmol TE)/g d.m. was used to express the results.

#### 2.5.2. Anti-Inflammatory Activity

The anti-inflammatory activity of fresh-b and dried red cabbage extracts was determined by a topical in vivo assay as described by Valenzuela-Barra et al. (2015) [48]. Adult male CF-1 mice (20–25 g), sourced from a stock at the ISP (Instituto de Salud Pública, Santiago, Chile), were used in the experiments. Two pro-inflammatory agents, phorbol 12-myristate 13-acetate (TPA) and arachidonic acid (AA), were used to check for possible anti-inflammatory pathways in the samples. Indomethacin and nimesulide were used as reference drugs against TPA and AA, respectively. The experimental setup involved treating mice with red cabbage extracts (3 mg/ear) along with TPA (5 μg in 20 μL acetone) or AA (2 mg in 20 μL acetone). Control mice were administered only AA or TPA with equivalent concentrations. The extracts and pro-inflammatory agents were applied to both the inner and outer surfaces of the right ear (10 μL each side), while the left ear received only acetone. Mice were euthanized by cervical dislocation (6 h after TPA application and 1 h after AA application). Then, a 6 mm diameter section of both the right and left ears was cut and weighed. The topical anti-inflammatory effect (*EA*) was assessed using the following equation:

(3) $$\%EA=\frac{W_{c}-W_{s}}{W_{c}}\times 100$$

where *W_C_* and *W_S_* stand for the median value difference of the weights of the treated animals’ right and left ear parts, respectively. This study was in accordance with the current “International Norms for the Biomedical Investigation with Animals” and received approval from the bio-ethics norms of Comité Institucional de Cuidado y Uso de Animales (CICUA) of the Universidad de Chile (certificate number: 22637—MED—UCH; date: 25 November 2022).

#### 2.5.3. Antiproliferative Assay

The antiproliferative effect of red cabbage extracts on gastric cancer cells was evaluated according to Macuer-Guzmán et al. (2019) [49]. The human gastric cell line (AGS) was grown and maintained in F-12K medium. The human gastric epithelial cell line (GES1) was cultured and maintained in DMEM (Corning^®^, Corning, NY, USA). Medium was used as a control for healthy cells. Both culture media (F-12K and DMEM) were supplemented with 10% FBS, and two cell lines (AGS and GES1) were maintained in a humidified atmosphere using a CO_2_ incubator (Shel Lab, Cornelius, OR, USA) at 37 °C with 5% CO_2_ and 95% humidity in the presence of 1% antibiotics (10,000 U/mL of penicillin and 10,000 µg/mL of streptomycin) (Corning^®^, Corning, NY, USA). Cells were seeded in a 96-well microplate and grown for 24 h until treatment with red cabbage extracts. The extract samples were filtered (0.2 µm) and performed at different concentrations, 32.5, 40 and 45 mg/mL, based on previous experiments. After 48 h of treatment, MTS solution was added to each well and incubated for 4 h at 37 °C. Absorbance was measured at 490 nm wavelength. Results were expressed as % cell viability.

### 2.6. Statistical Analysis

Using the Rstudio program (V. 1.4.1717), the results were statistically analyzed and reported as means ± standard deviation. At the 5% probability level, the means were compared using an analysis of variance (ANOVA) and Tukey’s test to determine the significance of the main effects. 

## 3. Results and Discussions

### 3.1. Proximate Composition 

Table 2 depicts the proximate composition of both fresh-b and dehydrated red cabbage. The moisture content in the fresh-b sample was similar to that reported in the specialized literature [33] and decreased considerably with the application of drying methods, especially with vacuum drying and freeze drying. This observation has a direct effect on the water activity values (a_w_), registering between 0.33 and 0.46, which is ideal for inhibiting bacterial growth (<0.61) [50]. Protein, fiber and ash remain stable after drying process. However, the lipid concentration mainly increased with freeze drying and vacuum drying, reaching five times. This phenomenon could be related to cell wall rupture during the drying process mostly at low temperatures [51], which can lead to the release of intracellular lipids. Cell disruption has been found to increase lipid yield on microalgae and yeast [52,53]. 

### 3.2. Determination of Total Phenolic, Total Flavonoid and Total Anthocyanin Content

Table 3 shows the total phenolic content (TPC), total flavonoid content (TFC) and total anthocyanin content (TAC) for fresh-b and dehydrated red cabbage, the latter two being subclasses of phenolic compounds. Phenolic compounds are relevant as they show an antioxidant effect by donating a hydrogen atom and/or an electron to free radicals, causing the oxidation chain reaction to break down. The latter is responsible for cellular oxidative stress, causing different health problems such as cancer, cardiovascular diseases, atherosclerosis, neurological disorders and hypertension, among others [54]. TPC value of fresh-b red cabbage was very similar to the results reported by Tan et al. (2023) [12], which registered a value of 11.86 ± 0.06 mg/g d.m. CD and IRD processes decreased TPC value slightly, and this phenomenon may be related to the instability of TPC at high temperatures, where the release of bound phenolic compounds, partial lignin degradation leading to the release of phenolic acid derivatives, and thermal degradation take place [55]. On the other hand, TPC remained relatively stable in the VD, LTVD and FD processes.

Moreover, flavonoids are secondary plant metabolites with a general structure combining two benzene rings with three carbons [56] and are very abundant in foods such as fruits or vegetables and some seeds, providing color, fragrance and flavor characteristics [57]. Numerous studies have been carried out to describe different health-promoting activities where the action of diverse types of flavonoids is involved. These activities include antioxidant, antiaging, anti-inflammatory and immunomodulatory activity, cardioprotective effects, and antimicrobial and antiviral potential, among others [58]. TFC values in fresh-b red cabbage have been reported in a range of 12.33 ± 0.11 to 17.44 ± 0.11 mg QE/g d.m. for aqueous and methanolic extracts, respectively [59]. These results are relatively close to the values obtained here, though the difference can be attributed to environmental and crop factors such as climate, soil quality and ripening degree. Convective drying has a notorious effect on TFC, decreasing by 37% with respect to the fresh-b value, probably caused by thermal degradation. This loss in total flavonoid content could be related to its molecular structure composed of benzene ring and hydroxyl groups (-OH), where these groups tend to oxidize, forming aldehydes, ketone groups or carboxyl groups when heated. In addition, they can be degraded by oxidation reactions, due to the hot air that passes through the drying chamber [60]. On the other hand, for FD and VD, the TFC remained relatively stable. 

Finally, anthocyanins are naturally occurring flavonoid plant pigment molecules that give many fruits and vegetables their blue, red or purple hues. These compounds have been described to have antioxidant and antimicrobial properties [61] and have been associated with the prevention of cardiovascular pathologies and neurodegenerative disorders [62]. Anthocyanin content in fresh-blanched red cabbage was similar to the content reported by Bernstein and Noreña (2017) [63], where TAC depended on water temperature and exposition time. The observed fresh-b value was similar to the values reported by Sakulnarmrat et al. (2021) [64] in red cabbage with 31.65 and 42.26 mg/100 g d.m. Additionally, the drying processes exerted degradation on these types of components, being more accentuated in CD and IRD methods, although VD, LTVD and FD showed losses with respect to the value of the fresh-b extract, since these are less aggressive than CD and IRD processes. The TPC, TFC and TAC data show that the degradation of these bio-compounds is less affected by processes like VD, LTVD and FD. These processes align with what is actually happening in an oxygen-free controlled environment, which avoids oxidation reactions with the bioactive compounds, allowing for greater stability and/or retention [65]. 

### 3.3. Determination of Total Glucosinolate Content (TGC)

Glucosinolates (GLS) are compounds found in plants, mainly in the *Brassicaceae* family. These compounds are widely recognized to have beneficial effects for human health, due to their derivatives produced by myrosinase enzymatic action. These compounds, known as epithionitriles, nitriles, thiocyanates and particularly isothiocyanates [66], have been reported to exhibit anti-inflammatory, antimicrobial, neuroprotective and cardioprotective properties, among others [67]. Table 3 shows the total glucosinolate content for fresh-blanched and dehydrated samples. Fresh-b TGC values are similar to previous results by convective drying at 60 °C with 44.7 ± 0.63 µmol SE/g d.m. [68]. The blanching process along with the drying process causes the myrosinase enzyme inactivation, allowing the GLS concentration to remain relatively stable [69]. However, the results in Table 3 show a lower TGC in the LTVD-dehydrated samples. Probably, during this process, a proportion of the enzyme remained active, degrading GLS; this should be reflected in a higher concentration of isothiocyanates. Moreover, IRD and CD processes also decrease TGC; this effect might be caused by thermal degradation. According to Oerlemans et al. (2006) [70], GLS such as glucoiberin, gluconapin, progoitrin, 4-hydroxyglucobrassicin, sinigrin, glucobrassicin, glucoraphanin and 4-methoxyglucobrassicin are thermolabile compounds and their concentrations decrease when heat treatment is applied. 

Nevertheless, research has demonstrated that myrosinase activity can be produced by the human gut microbiota, allowing GLS to be hydrolyzed, and it has even been reported that GLS and its derivatives can influence the composition of the gut microbiota [66]. Thus, foods with a high GLS content, such as red cabbage, have an important positive effect on human health.

### 3.4. Determination of Phenolic Acid Profile

Phenolic compounds are frequently found in many plant species, with hydroxycinnamic acids (HCAs) being the most predominant phenols in the *Brassica oleracea* plant. The group of HCAs comprises highly polar compounds that are generally attached to the cell wall of the plant [71]. This group of phenolic acids has been shown to have pharmacological properties attributed to the functions of the presence of hydroxyl groups linked to an aromatic ring [72,73,74], mainly high-power antioxidants. In red cabbage dehydrated by different methods, three types of hydroxycinnamic acids were identified (Table 3) and the chromatogram can be reviewed in Supplementary Materials. Ferulic acid was the most abundant in all the samples analyzed, followed by sinapic acid and caffeic acid; these results agree with the findings reported by other authors in red cabbage [39,72,75]. The drying methods, as well as the conditions used, had a significant influence on the HCA content compared to fresh-b samples. CD, VD and IRD methods had no significant difference (*p* < 0.05), and the total content of HCAs was 4611, 4414 and 4580 µg/g d.m., respectively (73% more than in fresh-b samples), while samples dried at low temperatures demonstrated a decrease in the total HCA content of approximately 23% with respect to thermal drying methods. The higher concentration of HCAs in thermal methods can be explained by the accumulation of phenolic compounds in vacuoles and other cellular structures. The use of high temperatures during the drying process releases phytochemicals bound to the matrix, facilitating the extraction of these compounds [12,76].

### 3.5. Determination of Sulforaphane Content

Sulforaphane is the major isothiocyanate in brassica vegetables, especially in broccoli, obtained from glucoraphanin hydrolysis by myrosinase [77]. It has been reported that this isothiocyanate can decrease the risk of developing breast cancer, gastric cancer and skin cancer [78]. Moreover, it has also been described as having antidiabetic/anti hyperlipidemic, anti-angiogenic, antioxidant, neuroprotective, anti-aging, cardioprotective, anti-inflammatory and antimicrobial activities [77]. Low concentrations of sulforaphane were registered in all samples (Table 3), which was expected due to myrosinase inactivation by the blanching process. However, the major SFN concentrations were found in the samples dehydrated by LTVD. We assume that a small fraction of the myrosinase enzyme remained active in the LTVD sample, due to the low-temperature drying process, hydrolyzing a proportion of glucosinolates and transforming them into their derivatives. This is consistent with a lower concentration of GLS in this same type of sample, as shown in Section 3.3.

### 3.6. Determination of Amino Acid and Fatty Acid Profile

Amino acids have an important role in regulatory functions in cells, nutrition and body homeostasis. Amino acids have been shown to play a role in the secondary metabolism process in plants, particularly in the synthesis of glucosinolates and phenolic compounds, which are crucial for both human health and plant–environment interactions [79]. Table 4 shows the amino acid content in red cabbage dehydrated by different methods. The amino acid content recorded in red cabbage was found within the ranges reported in green cabbage by Lin et al. (2021) [80], and similar to our results where a major part with the presence of glutamic acid was observed. Glutamic acid is very important at the nutritional level, because it has been reported to play a role as an antioxidant agent [81]. Aspartic acid was also found in higher proportion compared to the other amino acids. This amino acid has been categorized as a functional amino acid (FAA) due to its role as a precursor to methionine, threonine, isoleucine, and lysine, and regulates hormone secretion [82]. When comparing drying technologies, the total content of essential amino acids (EAAs) was found to be influenced by the specific drying method employed, with an increase mainly in the FD- and IRD-treated samples. This phenomenon could be related to the fact that red cabbage has been reported to have a high content of soluble sugars [83], which can interact with proteins during the IRD process, generating a Maillard reaction, releasing free amino acids from protein [84]. A similar trend was observed for total amino acid content.

Also, fatty acids are the main components of complex lipids and fat acquired in the human diet. The fatty acids consumed in the diet have general and specific biological activities that influence the metabolism, function and responsiveness of cells. Therefore, they influence health, well-being or the risk of disease [85]. Table 5 depicts the fatty acid profile found in dehydrated red cabbage, where the total content of saturated fatty acids (SFAs), monounsaturated fatty acids (MUFAs) and polyunsaturated fatty acids (PUFAs) is also described. In saturated fatty acids, palmitic acid was predominant, followed by stearic acid, which have been related to different cardiovascular diseases, cancer and diabetes [86]. However, the main proportion of fatty acids corresponds to the group of polyunsaturated acids with a higher concentration of γ-linolenic acid (GLA), followed by linoleic acid (LA); both are essential and must be supplied by the diet. γ-Linolenic acid has three double bonds, belonging to the n-6 family of fatty acids. It performs biologically important functions such as acting as a substrate for the synthesis of eicosanoids, participating in the transport and oxidation of cholesterol and being one of the components of the lipid membrane [87]. In addition, GLA has now been reported to have anti-inflammatory effects, having potential for the development of therapeutics for the treatment of polycystic ovary syndrome-associated pro-inflammatory response in humans [88]. In this way, eating foods high in GLA, like red cabbage, is beneficial to human health and metabolism. On the other hand, it has been reported that the main beneficial effect of linoleic acid is that it contributes to reducing low-density lipoprotein (LDL) cholesterol concentrations by replacing SFA in the diet, having a correlation with a decreased risk of cardiovascular pathologies [85]. According to the total SFAs, MUFAs and PUFAS of dried samples, the fatty acid content remained relatively stable regardless of the drying technology used. 

### 3.7. Health-Promoting Properties

#### 3.7.1. Antioxidant Potential by DPPH and ORAC Assays

**Figure 1:**
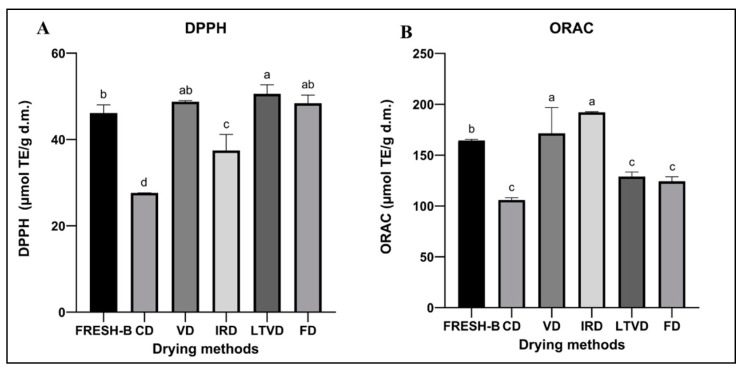
Antioxidant activity for samples extracts from fresh-blanched and dehydrated red cabbage. FRESH-B: fresh-blanched; VD: vacuum drying; CD: convective drying; IRD: infrared drying; LTVD: low-temperature vacuum drying; FD: freeze drying. Values are expressed as mean ± standard deviation. (**A**) is DPPH assay and (**B**) is ORAC assay. Different letters indicate significant differences (*p* < 0.05).

Oxygen free radicals facilitate the oxidation process in the human body, which leads to a decrease in metabolic performance [89]. The molecular pathways’ oxidative stress response includes DNA damage, genetic mutations, fatty acid oxidation, changes in protein function, proteins in live cells and apoptosis promotion [90]. According to reports, oxidative stress plays a role in the pathophysiology of lung diseases (chronic pulmonary obstruction and lung cancer), neurological disorders, cardiovascular diseases, diabetes, bipolar disorder, depression, renal disease and aging [91]. Conversely, antioxidants are chemical molecules that, although present in smaller quantities than an oxygen electrode, have the ability to postpone or stop oxidation by preventing other compounds from disintegrating during the oxidation process. Since the body’s natural first line of defense against free radicals is antioxidants, they are extremely important [92]. Antioxidants present in food, especially fruits and vegetables, are crucial because they may be added to food as supplements and have a protective impact against certain diseases, including cancer and heart disease [93]. In this sense, red cabbage is an interesting source of bio-compounds due to its fresh-b and dehydrated samples, which show antioxidant potential as evaluated by DPPH and ORAC assays. The fresh-blanched red cabbage sample showed an antioxidant capacity, measured by DPPH assay, of 46.14 ± 1.86 µmol TE/g d.m. (Figure 1A), which was a similar value to that reported by Wiczkowski et al. (2015) [94] for fresh-b red cabbage (47.0 ± 1.0 µmol TE/g d.m.). Drying methods such as CD and IRD exert a clear decreasing effect on antioxidant capacity as determined by DPPH. This phenomenon could be caused by the thermal degradation of thermosensitive phytochemicals and oxidation reactions due to oxygen content in the drying chamber [95]. Drying with vacuum technology (VD, LTVD and FD) maintained the antioxidant capacity at a relatively stable level, and even higher DPPH activity was observed, mainly in LTVD. This phenomenon may be connected to the production of some phytochemicals, including flavones, flavonols and flavonoids [96]. On the other hand, the ORAC assay showed differences (Figure 1B) due to the different oxidation reaction mechanism. Fresh-b sample showed 164.25 ± 1.2 µmol TE/g d.m. activity and better retention of antioxidant activity was observed in the samples dehydrated by VD. These results are in agreement with Karam et al. (2016) [97], where vacuum drying was an appropriate method for protecting not only phenolic compounds and anthocyanins in fruits and vegetables, but also to preserve the antioxidant activity of these compounds. Interestingly, the IRD method obtained a higher antioxidant activity, even higher compared to the fresh-b sample. It has been reported that the application of infrared radiation can improve nutrient retention, total phenolic content, flavonoid content, antioxidant activity and vitamin C [30]. CD showed the lowest retention of antioxidant activity; this could be associated with the heat treatment and oxidative reaction that cause the degradation of phenolic compounds during the drying process. Based on the results, vacuum drying demonstrated a better retention of bio-compounds, primarily attributed to the absence of oxygen inside the chamber, which prevents the oxidation of antioxidants and preserves total phenolic content (TPC) and anthocyanins. This preservation can contribute to its health-promoting properties, especially antioxidant potential. From a physiological point of view, vacuum drying represents a less aggressive drying process due to its low-oxygen environment, which aids in maintaining the integrity of antioxidant compounds [98]. Red cabbage dehydrated under low-oxygen environmental conditions could be considered as a functional food ingredient due to the observed antioxidant activity. However, further studies concerning the action mechanism and bioaccessibility would need to be applied in future works.

#### 3.7.2. Anti-Inflammatory Activity

**Figure 2:**
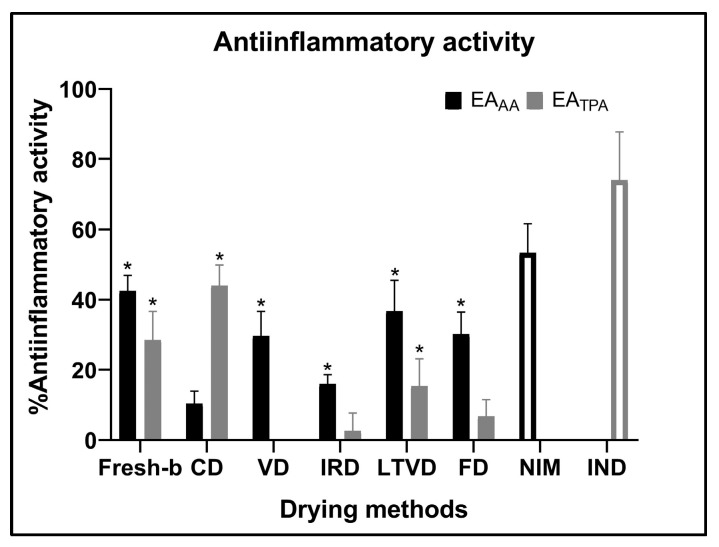
Anti-inflammatory activity of red cabbage extracts dehydrated by different drying methods. VD: vacuum drying; CD: convective drying; IRD: infrared drying; LTVD: low-temperature vacuum drying; FD: freeze drying. EA_TPA_: topical anti-inflammatory effect against phorbol 12-myristate 13-acetate; EA_AA_: topical anti-inflammatory effect against arachidonic acid. NIM: nimesulide; IND: indomethacin as control medicament. n.d: not determined. An asterisk (*) denotes significant differences (*p* < 0.05) between samples with respect to the negative control (100% inflammation); n = 8.

The protective mechanism that organisms display against harmful stimuli is called inflammation, and it is characterized by an increased blood flow to the injured tissue, which causes pain, heat, swelling, redness and the damaged part to become non-functional [99]. This inflammation can be categorized as chronic or acute, systemic or localized, and can be produced by different mechanisms through various types of mediators, including prostaglandins, cytokines and reactive oxygen species [100]. The latter causes various diseases, including chronic inflammation-associated disorders [101]. The development of natural, safe and effective anti-inflammatory agents is extremely essential for the development of therapies for different diseases, such as some neurodegenerative pathologies (multiple sclerosis, Alzheimer’s disease and Parkinson’s disease) and metabolic disorders (atherosclerosis, type 2 diabetes and obesity) [102]. Figure 2 depicts the anti-inflammatory activity of dehydrated red cabbage extracts was tested by two different models: 12-O-tetradecanoylphorbol-13-acetate (TPA) and arachidonic acid (AA), and two reference drugs: indomethacin and nimesulide. Indomethacin and nimesulide were applied on the respective inflammatory model in rats, showing a 74% (0.5 mg/ear TPA inflammation) and 53.4% (1.0 mg/ear AA inflammation) reduction in ear edema. In both models, the respective references were more effective in reducing the level of inflammation generated. Between the samples analyzed, the fresh-blanched sample exerted the best anti-inflammatory activity in the AA model with a 42% ear edema reduction. The AA model generates rapid inflammation and is produced by an increase in myeloperoxidase and elastase activity due to the approach of neutrophils after the application of this inflammatory agent [103]. The higher content of anthocyanins, ferulic acid and polyphenols of the fresh-b extract might explain the capacity to counteract the causal effect of inflammation in the AA model [104]; these components could be affected during the dehydration process, although to a lesser extent in LTVD, FD and VD. Probably, the low oxygen level in the drying chamber of these methods allows the best retention of anti-inflammatory components able to hinder the effect of the AA model. The TPA model activates the protein kinase C, triggering chemokine formation through epidermal cells and thus the infiltration of neutrophils, which release enzymes as myeloperoxidase and free radicals as reactive oxygen species that contribute to host defense [105]. In this model, CD technology had the best anti-inflammatory activity with a 44% inflammation reduction. The primary phenolic component found in the red cabbage extracts, and particularly in the sample dehydrated using convective drying, was ferulic acid. Some authors have reported on the anti-inflammatory potential of ferulic acid by reducing the intensity of pro-inflammatory mediators such as prostaglandin E2, tumor necrosis factor alpha and iNOS (inducible nitric oxide synthase) capacity [104]. It has also been reported that ferulic acid inhibits neuroinflammation in an in vivo model, decreasing pro-inflammatory cytokines in the prefrontal cortex and is also closely related to an antidepressant effect [106]. Considering this background, ferulic acid is probably involved in the anti-inflammatory effect observed in the TPA model. Despite the anti-inflammatory activity detected in red cabbage, further studies on its anti-inflammatory potential could be carried out using other models to investigate the mechanism of action.

#### 3.7.3. Antiproliferative Activity

**Figure 3:**
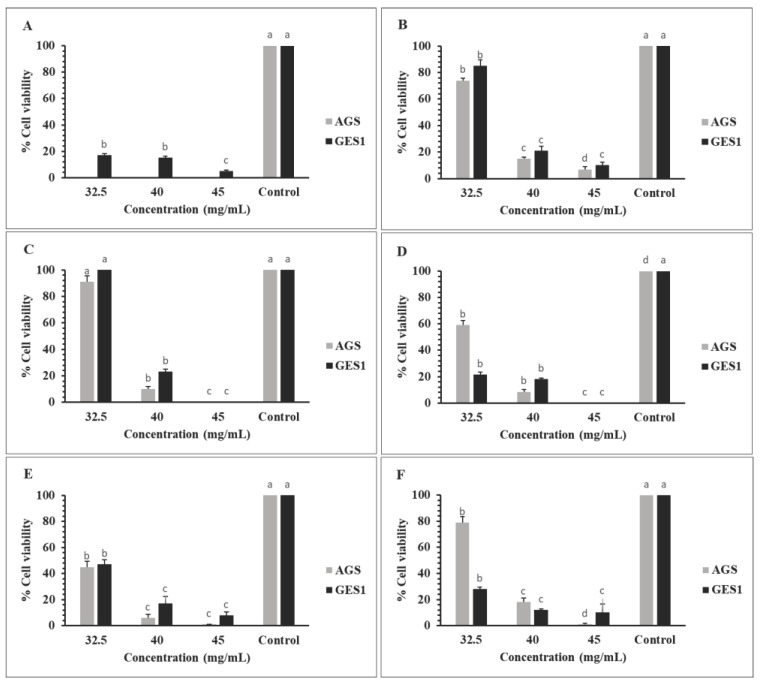
Antiproliferative activity of fresh-b and dehydrated red cabbage extracts (mg/mL) on gastric cancer cells. (**A**) Fresh-blanched, (**B**) CD: convective drying, (**C**) IRD: infrared draying, (**D**) VD: vacuum drying, (**E**) LTVD: low-temperature vacuum drying and (**F**) FD: freeze drying. Control: without red cabbage extract. The statistical analysis of AGS and GES1 was evaluated separately. Different letters indicate significant differences (*p* < 0.05).

Cancer involves a broad spectrum of diseases characterized by abnormal and uncontrolled cell proliferation in organs or tissues, leading to an invasive behavior of cells to other parts of the body [107]. The genesis of most cancers is attributed to the interaction between DNA and carcinogens or reactive oxygen species (ROS), resulting in the production of mutated DNA copies. This explains the relevance of antioxidant activity to scavenging free radicals, binding metal ions and regulating cell signaling pathways [108]. Vegetable extracts are able to stabilize reactive oxygen species due to free radical scavenging capacity of antioxidants. These modulations lead to the protection of cellular lipids, proteins and DNA from molecular damage [109]. The antiproliferative activity of fresh-b and dehydrated red cabbage extracts at different concentrations (32.5, 40 and 45 mg/mL) was measured in the human gastric cell line of atrophic gastritis (AG–gastric cancer model) with the human gastric epithelial cell line (GES1) as healthy cells. Figure 3A shows the antiproliferative activity of fresh-b red cabbage extract, where all concentrations inhibit the growth of cancer cells. However, healthy cells are also affected, although to a lesser degree. The observed effect on AGS cells could be associated with the presence of anthocyanins, which are indeed more concentrated in the fresh-b sample. It has been described that anthocyanin-rich extracts have antiproliferative activity [110], and especially in gastric cancer cell models [111]. On the other hand, the dried samples showed an antiproliferative effect, which was more pronounced as the concentration of red cabbage extract increased, also affecting healthy cells. However, the LTVD extract at 40 mg/mL showed the highest efficiency against cancer cells, decreasing it by 94%, which is higher than the effect observed in healthy cells. The concentrated extract at 45 mg/mL proved to be too aggressive for both line cells and the concentration at 32.5 mg/mL was the least efficient. Interestingly, samples dehydrated by LTVD showed a higher sulforaphane content. This compound has been described as having an anticarcinogenic effect [112]; therefore, it could be the main factor responsible for the activity observed in this red cabbage extract. The action mechanism of sulforaphane on gastric cancer cells is not easy to elucidate. Nevertheless, it has been reported that the action against AGS cells takes place through the promotion of apoptosis, the latter by mitochondrial apoptotic signaling. This apoptotic induction is accompanied by ROS production and increased AMPK (AMP (adenosine monophosphate)-activated protein kinase) activation [113]. These findings establish a basis for further in vivo studies to achieve a comprehensive insight related to the antiproliferative activity of red cabbage.

## 4. Conclusions

In this work, the effect of different drying methods on the retention of bio-compounds and health-promoting properties were evaluated. It was determined that fresh-b red cabbage presents bio-compounds such as polyphenols, including flavonoids and anthocyanins, with a higher retention of these compounds after being dehydrated by the LTVD method, due to the low temperatures and low oxygen level in the drying chamber. The blanching and drying processes inactivate the myrosinase enzyme, keeping the glucosinolate content stable. However, LTVD showed lower TGC so that enzyme traces would remain active, which is reflected in the observed sulforaphane content. Three hydroxycinnamic acids were identified with high concentrations in the case of thermal processes, which facilitated their respective extractions. Dehydrated red cabbages were shown to be a rich source of fatty acids and amino acids, especially in the IRD-subjected sample. The antioxidant potential varied among the drying method used, mainly for IRD, which showed high antioxidant activity as measured by ORAC assay. Anti-inflammatory activity was observed in all extracts and models evaluated independent of the drying method, with a greater effect in the CD-dehydrated samples in the TPA model, probably due to the action of some bio-compounds such as ferulic acid. All red cabbage extracts showed an antiproliferative effect, mostly in the LTVD sample due to its sulforaphane content. Accordingly, the dehydration process is a great alternative for the preservation of red cabbage, allowing the retention of bio-compounds that generate health-promoting properties.

## Figures and Tables

**Table 1:** Drying conditions and equipment.

Drying Process	Equipment	Conditions
CD	Hot air dryer designed and constructed at the Department of Food Engineering, Universidad de La Serena, La Serena, Chile.	60 °C and set at 1.5 m/s air speed for 5.5 h.
VD	Vacuum oven (Memmert, model VO 400, Schwabach, Germany).	10 kPa and 60 °C for 10 h.
IRD	Infrared drying oven designed and constructed at the Department of Food Engineering, Universidad de La Serena, La Serena, Chile.	Two 175-Watt lamps as radiant source for 8.5 h at 60 °C.
LTVD	Vacuum oven (Memmert, model VOcool 400, Schwabach, Germany).	1 kPa and 20 °C for 35.6 h.
FD	Freeze-dryer (Virtis, AdVantage Plus, Gardiner, NY, USA).	Pre-frozen at −80 °C and then freeze-dried for 24 h. Condenser temperature: −60 °C and chamber pressure: 0.027 kPa.

**Table 2:** Proximate composition content and water activity of fresh-b and dehydrated red cabbage.

Parameters g/100 g d.m.	Fresh-b	CD	VD	IRD	LTVD	FD
Water *	92.59 ± 0.12 ^a^	14.05 ± 0.09 ^c^	11.31 ± 0.08 ^d^	13.99 ± 0.04 ^c^	15.06 ± 0.56 ^b^	10.95 ± 0.23 ^d^
Lipid	0.21 ± 0.01 ^e^	1.04 ± 0.03 ^b^	0.53 ± 0.07 ^d^	0.90 ± 0.04 ^c^	0.96 ± 0.09 ^bc^	1.15 ± 0.02 ^a^
Ash	9.99 ± 0.33 ^b^	8.02 ± 0.06 ^e^	8.22 ± 0.06 ^de^	8.36 ± 0.09 ^d^	10.27 ± 0.14 ^a^	9.00 ± 0.05 ^c^
Protein	19.19 ± 3.10 ^a^	17.87 ± 0.36 ^ab^	17.14 ± 0.27 ^ab^	17.32 ± 0.28 ^ab^	15.76 ± 0.30 ^b^	17.02 ± 0.16 ^ab^
Crude fiber	13.31 ± 0.61 ^a^	10.95 ± 1.19 ^b^	9.91 ± 0.95 ^b^	12.35 ± 0.41 ^ab^	13.15 ± 1.32 ^a^	11.53 ± 0.96 ^ab^
Total carbohydrates (difference)	72.01 ± 1.88 ^c^	62.80 ± 0.41 ^ab^	65.73 ± 0.23 ^ab^	63.15 ± 0.32 ^ab^	62.02 ± 0.81 ^abc^	64.86 ± 0.09 ^bc^
Water activity **	0.993 ± 0.004 ^a^	0.467 ± 0.002 ^b^	0.339 ± 0.003 ^e^	0.452 ± 0.002 ^c^	0.427 ± 0.001 ^d^	0.335 ± 0.01 ^e^

* Content value per 100 g fresh weight; ** dimensionless. Fresh-b: Fresh-blanched, CD: convective drying, VD: vacuum drying, IRD: infrared drying, LTVD: low-temperature vacuum drying and FD: freeze drying. Different letters in the same row indicate significant differences (*p* < 0.05).

**Table 3:** Bio-compound content of extracts of fresh-b and dehydrated red cabbage.

Parameters	Drying Methods					
	Fresh-b	CD	VD	IRD	LTVD	FD
TPC (mg GAE/g d.m.)	11.53 ± 0.39 ^ab^	9.81 ± 0.28 ^d^	11.89 ± 0.28 ^a^	9.93 ± 0.27 ^d^	10.91 ± 0.51 ^bc^	10.80 ± 0.34 ^c^
TFC (mg QE/g d.m.)	10.56 ± 0.75 ^b^	6.66 ± 0.59 ^d^	11.30 ± 0.90 ^a^	9.26 ± 0.39 ^c^	8.77 ± 0.67 ^c^	10.15 ± 0.62 ^b^
TAC (mg Cya3 glu/g d.m.)	0.391 ± 0.004 ^a^	0.196 ± 0.025 ^d^	0.265 ± 0.011 ^b^	0.122 ± 0.009 ^e^	0.257 ± 0.019 ^b^	0.223 ± 0.005 ^c^
Caffeic acid (µg/g d.m.)	ND	479 ± 20.08 ^a^	427 ± 10.80 ^ab^	388 ± 29.04 ^b^	412 ± 28.72 ^b^	290 ± 44.38 ^c^
Ferulic acid (µg/g d.m.)	1520 ± 69.24 ^d^	3173 ± 11.40 ^a^	3073 ± 70.21 ^a^	3118 ± 242.66 ^a^	2568 ± 153.86 ^b^	2151 ± 334.86 ^c^
Sinapic acid (µg/g d.m.)	1107 ± 85.60 ^c^	959 ± 10.03 ^bc^	915 ± 19.06 ^cd^	1075 ± 74.62 ^ab^	795 ± 44.43 ^c^	785 ± 102.57 ^c^
TGC (µmol SE/g d.m.)	47.38 ± 2.32 ^b^	43.26 ± 3.76 ^c^	51.15 ± 3.31 ^a^	42.17 ± 1.95 ^c^	41.30 ± 3.37 ^c^	47.05 ± 3.05 ^b^
SFN (mg/g d.m.)	ND	0.004 ± 0.002 ^b^	0.039 ± 0.004 ^b^	0.001 ^d^	0.070 ± 0.004 ^a^	0.032 ± 0.001 ^c^

VD: vacuum drying; CD: convective drying; IRD: infrared drying; LTVD: low-temperature vacuum drying; FD: freeze drying. Values are expressed as mean ± standard deviation. Different letters in the same row indicate significant differences (*p* < 0.05). Standard deviation was calculated on three replicates. Total anthocyanin content (TAC); total phenolic content (TPC); total flavonoid content (TFC); total glucosinolate content (TGC); sulforaphane content (SFN); gallic acid equivalents (GAE); quercetin equivalents (QE); sinigrin equivalents (SE). ND: not detected.

**Table 4:** Amino acid profile in red cabbage dehydrated by different drying methods.

Amino Acids (g/100 g) d.m.	CD	VD	IRD	LTVD	FD
Aspartic acid	0.83 ± 0.08 ^b^	0.64 ± 0.11 ^bc^	1.06 ± 0.04 ^a^	0.47 ± 0.14 ^c^	1.19 ± 0.18 ^a^
Glutamic acid	2.99 ± 0.29 ^a^	2.10 ± 0.04 ^b^	3.37 ± 0.10 ^a^	1.13 ± 0.34 ^c^	3.58 ± 0.51 ^a^
Serine	0.59 ± 0.03 ^ab^	0.47 ± 0.09 ^b^	0.69 ± 0.03 ^a^	0.26 ± 0.08 ^c^	0.67 ± 0.11 ^a^
Glycine	0.46 ± 0.06 ^b^	0.40 ± 0.11 ^b^	0.60 ± 0.01 ^ab^	0.17 ± 0.07 ^c^	0.47 ± 0.07 ^a^
Threonine ^1^	0.29 ± 0.05 ^bc^	0.24 ± 0.07 ^b^	0.44 ± 0.03 ^a^	0.15 ± 0.05 ^c^	0.47 ± 0.09 ^c^
Arginine	0.72 ± 0.04 ^ab^	0.58 ± 0.16 ^b^	0.84 ± 0.01 ^a^	0.30 ± 0.08 ^c^	0.74 ± 0.12 ^ab^
Alanine	0.47 ± 0.04 ^a^	0.35 ± 0.08 ^b^	0.52 ± 0.01 ^a^	0.21 ± 0.06 ^c^	0.54 ± 0.08 ^a^
Tyrosine	0.24 ± 0.00 ^b^	0.29 ± 0.02 ^ab^	0.32 ± 0.04 ^a^	0.04 ± 0.02 ^c^	0.26 ± 0.07 ^ab^
Valine ^1^	0.31 ± 0.04 ^b^	0.28 ± 0.05 ^b^	0.45 ± 0.02 ^a^	0.17 ± 0.05 ^c^	0.45 ± 0.06 ^a^
Phenylalanine	0.44 ± 0.01 ^b^	0.37 ± 0.10 ^bc^	0.56 ± 0.01 ^a^	0.28 ± 0.10 ^c^	0.61 ± 0.08 ^a^
Isoleucine ^1^	0.68 ± 0.04 ^bc^	0.58 ± 0.09 ^c^	0.90 ± 0.04 ^a^	0.36 ± 0.11 ^d^	0.83 ± 0.12 ^ab^
Leucine ^1^	0.30 ± 0.00 ^b^	0.29 ± 0.08 ^b^	0.41 ± 0.01 ^a^	0.18 ± 0.04 ^c^	0.30 ± 0.06 ^b^
Lysine ^1^	0.44 ± 0.01 ^bc^	0.37 ± 0.04 ^c^	0.52 ± 0.03 ^ab^	0.22 ± 0.07 ^d^	0.60 ± 0.07 ^a^
Total EAAs	2.02 ± 0.15 ^b^	1.76 ± 0.33 ^b^	2.73 ± 0.13 ^a^	1.08 ± 0.32 ^c^	2.65 ± 0.41 ^a^
Total AA	8.76 ± 0.69 ^ab^	6.9 ± 1.47 ^b^	10.70 ± 0.37 ^a^	4.0 ± 1.20 ^c^	10.70 ± 1.61 ^a^

^1^ Total essential amino acids: Thr, Val, Met, Ile, Leu, His and Lys (excluding Trp). VD: vacuum drying; CD: convective drying; IRD: infrared drying; LTVD: low-temperature vacuum drying; FD: freeze drying. Values are expressed as mean ± standard deviation. Different letters in the same row indicate significant differences (*p* < 0.05).

**Table 5:** Fatty acid profile in dehydrated red cabbage by different drying methods.

Fatty Acid (g/100 g FAMES)	Drying Methods				
	CD	VD	IRD	LTVD	FD
Saturated Fatty Acids					
C12:0 Lauric Acid	0.07 ± 0.02 ^a^	0.17 ± 0.04 ^a^	0.07 ± 0.04 ^a^	0.13 ± 0.05 ^a^	0.03 ± 0.01 ^a^
C14:0 Myristic Acid	0.11 ± 0.01 ^b^	0.29 ± 0.03 ^a^	0.07 ± 0.02 ^b^	0.03 ± 0.01 ^b^	0.09 ± 0.04 ^b^
C15:0 Pentadecanoic Acid	0.40 ± 0.06 ^ab^	0.52 ± 0.06 ^a^	0.33 ± 0.01 ^b^	0.29 ± 0.04 ^b^	0.42 ± 0.06 ^ab^
C16:0 Palmitic Acid	15.95 ± 0.37 ^b^	15.90 ± 0.36 ^b^	17.44 ± 0.39 ^a^	16.92 ± 0.513 ^ab^	17.39 ± 0.15 ^a^
C17:0 Heptadecanoic Acid	0.57 ± 0.02 ^c^	0.87 ± 0.06 ^a^	0.79 ± 0.02 ^b^	0.65 ± 0.02 ^c^	0.61 ± 0.02 ^c^
C18:0 Stearic Acid	4.81 ± 0.17 ^a^	3.99 ± 0.14 ^b^	4.13 ± 0.11 ^b^	3.95 ± 0.14 ^b^	4.38 ± 0.25 ^ab^
C20:0 Arachidic Acid	0.59 ± 0.09 ^a^	0.59 ± 0.02 ^b^	0.40 ± 0.07 ^b^	0.35 ± 0.04 ^b^	0.56 ± 0.04 ^ab^
C22:0 Behenic Acid	0.28± 0.14 ^a^	0.56 ± 0.25 ^a^	0.19 ± 0.07 ^a^	0.21 ± 0.06 ^a^	0.35 ± 0.02 ^a^
C23:0 Tricosanoic Acid	n.d.	n.d.	n.d.	n.d.	0.69 ± 0.14
Monounsaturated fatty acids					
C16:1 Palmitoleic Acid	0.28 ± 0.07 ^ab^	0.43 ± 0.08 ^a^	0.21 ± 0.03 ^b^	0.17 ± 0.05 ^b^	0.25 ± 0.07 ^ab^
C17:1 Cis-10-Heptadecenoic Acid	0.32 ± 0.03 ^a^	n.d.	0.29 ± 0.01 ^a^	0.35 ± 0.02 ^a^	0.32 ± 0.07 ^a^
C18:1n9c/C18:1n9t Oleic Acid/Elaidic Acid	1.56 ± 0.01 ^a^	1.31 ± 0.09 ^b^	1.21 ± 0.03 ^bc^	1.06 ± 0.07 ^c^	1.56 ± 0.08 ^a^
C20:1n9 Cis-11-Eicosenoic Acid	0.31 ± 0.21 ^a^	0.16 ± 0.02 ^a^	0.11 ± 0.04 ^a^	0.16 ± 0.03 ^a^	0.29 ± 0.08 ^a^
C24:1n9 Nervonic Acid	0.43 ± 0.08 ^ab^	0.70 ± 0.24 ^a^	0.41 ± 0.03 ^ab^	0.34 ± 0.11 ^b^	0.47 ± 0.04 ^ab^
Polyunsaturated Fatty Acids					
C18:2n6c Linoleic Acid	23.43 ± 0.12 ^b^	21.69 ± 0.37 ^c^	21.69 ± 0.07 ^c^	22.19 ± 0.10 ^c^	24.46 ± 0.11 ^a^
C18:3n6 γ-linolenic acid	50.04 ± 0.17 ^ab^	51.61± 1.73 ^a^	51.97 ± 0.12 ^a^	52.33 ± 0.16 ^a^	47.51 ± 0.56 ^b^
C20:2 Cis-11,14-Eicosadienoic Acid	0.15 ± 0.05 ^a^	0.39 ± 0.21 ^a^	0.16 ± 0.09 ^a^	0.25 ± 0.08 ^a^	0.27 ± 0.06 ^a^
C20:3n3 Cis-11,14,17-Eicosatrienoic Acid	0.14 ± 0.03 ^a^	0.46 ± 0.24 ^a^	0.11 ± 0.07 ^a^	0.14 ± 0.01 ^a^	0.18 ± 0.02 ^a^
C22:6n3 Cis-4,7,10,13,16,19-Docosahexaenoic Acid	0.64 ± 0.26 ^a^	1.07 ± 0.55 ^a^	0.42 ± 0.10 ^a^	0.40 ± 0.05 ^a^	n.d.
SFAs	22.79 ± 0.23 ^bc^	22.90 ± 0.22 ^bc^	23.41 ± 0.22 ^b^	22.53 ± 0.34 ^c^	24.51 ± 0.29 ^a^
MUFAs	2.80 ± 0.192 ^a^	2.90 ± 0.52 ^a^	2.22 ± 0.12 ^a^	2.08 ± 0.21 ^a^	2.79 ± 0.14 ^a^
PUFAs	74.40 ± 0.11 ^a^	74.58 ± 0.90 ^a^	74.36 ± 0.13 ^a^	75.17 ± 0.35 ^a^	77.62 ± 0.43 ^b^

Values are expressed as mean ± sd of three replicated determinations. Values in the same raw with different superscript letters indicate significant differences (*p* < 0.05). FAMES (fatty acid methyl ester standard), SFAs (saturated fatty acids), MFAs (monounsaturated fatty acids), and PFAs (polyunsaturated fatty acids). n.d.: not detected.

## Data Availability

The original contributions presented in the study are included in the article/Supplementary Materials, further inquiries can be directed to the corresponding author.

## Supplementary Materials

The following supporting information can be downloaded at: https://www.mdpi.com/article/10.3390/foods13060830/s1.
