# Supplementary material for: Health-Promoting Properties of Processed Red Cabbage (Brassica oleracea var. capitata f. rubra): Effects of Drying Methods on Bio-Compound Retention
Source: Foods. 2024 Mar 8;13(6):830. doi: 10.3390/foods13060830 (PMC10969148; doi:10.3390/foods13060830)
Supplement: Supplementary file 1 [file foods-13-00830-s001.zip › foods-2883096-SI.pdf]

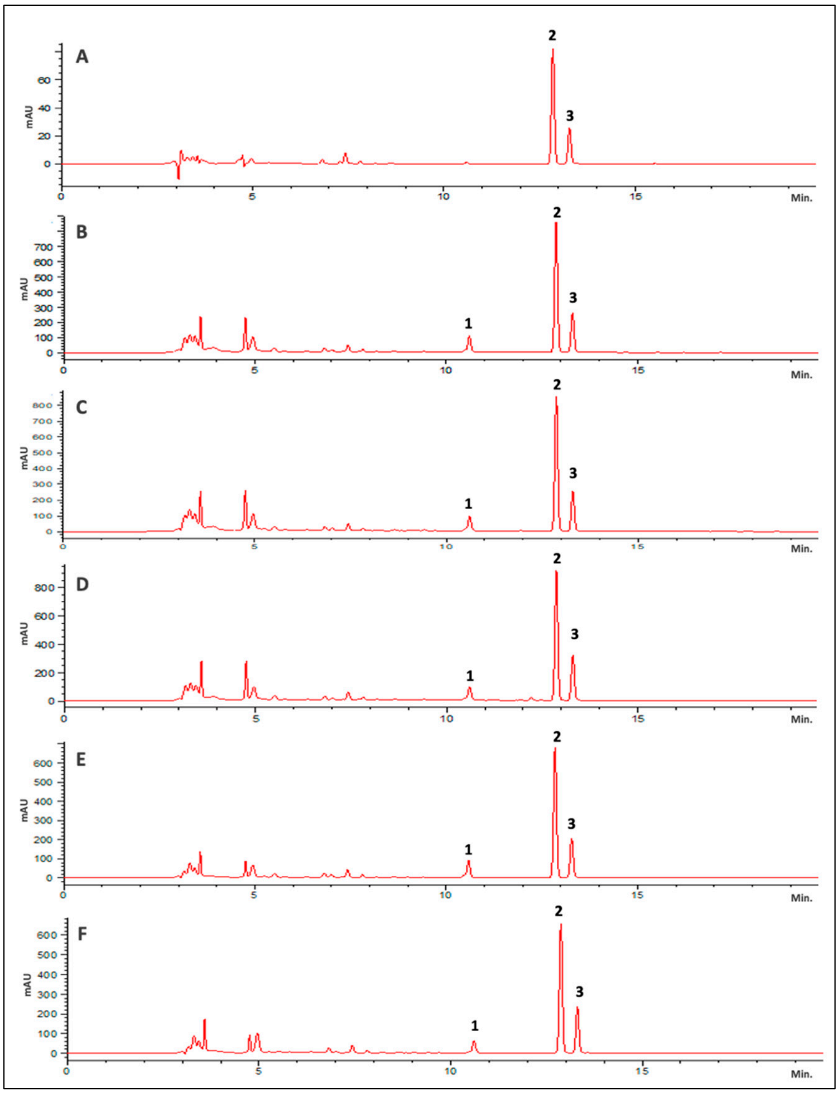

Supplementary figure: UV chromatogram (310 nm) of fresh-b and dried red cabbage by different drying methods. *(A) fresh-b, (B) hot air drying, (C) vacuum drying, (D) infrared drying, (E) low temperature vacuum drying, and (F) freeze-drying. Peaks: (1) Caffeic acid, (2) Ferulic acid, (3) Sinapic acid.*
